# The Usefulness of Coregistration with iFR in Tandem or Long Diffuse Coronary Lesions: The iLARDI Randomized Clinical Trial

**DOI:** 10.3390/jcm13154342

**Published:** 2024-07-25

**Authors:** Francisco Hidalgo, Rafael Gonzalez-Manzanares, Javier Suárez de Lezo, Ignacio Gallo, Marco Alvarado, Jorge Perea, Luis Carlos Maestre-Luque, Adriana Resúa, Miguel Romero, María López-Benito, Armando Pérez de Prado, Soledad Ojeda, Manuel Pan

**Affiliations:** 1 Department of Cardiology, Reina Sofia University Hospital, 14004 Cordoba, Spain; rafael.gonzalez@imibic.org (R.G.-M.); jslht@yahoo.es (J.S.d.L.); ignacio.gallo.sspa@juntadeandalucia.es (I.G.); jorge.perea.sspa@juntadeandalucia.es (J.P.); h42malul@uco.es (L.C.M.-L.); adriana.resua.sspa@juntadeandalucia.es (A.R.); maromero@uco.es (M.R.); soledad.ojeda.sspa@juntadeandalucia.es (S.O.); md1paalm@uco.es (M.P.); 2 Maimonides Biomedical Research Institute of Cordoba (IMIBIC), 14004 Cordoba, Spain; 3 Centro de Investigación Biomédica en Red Enfermedades Cardiovasculares (CIBERCV), 28029 Madrid, Spain; 4 Department of Medicine, University of Cordoba, 14004 Cordoba, Spain; 5 Department of Cardiology, University Hospital of Leon, 24008 Leon, Spainaperez@fundacionepic.org (A.P.d.P.)

**Keywords:** coronary artery disease, percutaneous coronary intervention, coronary physiology, iFR, randomized controlled trial

## Abstract

**Background**. Despite technical advancements, patients with sequential or diffuse coronary lesions undergoing percutaneous coronary intervention (PCI) have an increased risk of cardiovascular events at follow-up. We aimed to analyze the utility of a SyncVision/iFR (S-iFR)-guided PCI strategy versus an angiography-guided strategy in patients with this type of lesions. **Methods**. Randomized, multicenter, controlled, and open-label trial to compare S-iFR versus angiography-guided PCI in patients with sequential or diffuse angiographic coronary stenosis (ClinicalTrials.gov identifier: NCT04283734). The primary endpoint was the implanted stent length. The main secondary endpoint was targeting vessel failure (TVF) at one year. **Results**. A total of 100 patients underwent randomization, with 49 patients assigned to the S-iFR group and 51 to the angiography-guided PCI group. There were no differences between groups regarding clinical and anatomical characteristics. The baseline iFR was 0.71 ± 0.16 vs. 0.67 ± 0.19 (*p* = 0.279) in the S-iFR and angiography group, respectively. The mean lesion length was 42.3 ± 12 mm and 39.8 ± 12 (*p* = 0.297). The implanted stent length was 32.7 ± 17.2 mm in the S-iFR group and 43.1 ± 14.9 mm in the angiography group (mean difference, −10.4 mm; 95% confidence interval [CI], −16.9 to −4.0; *p* = 0.002). At one year, target vessel failure (TVF) occurred in four patients: three (6.1%) in the S-iFR group vs. one (1.9%) in the angiography group (*p* = 0.319). **Conclusions**. Among patients with sequential or long diffuse coronary lesions, a S-iFR-guided PCI strategy resulted in a reduction of the total stent length compared to an angiography-guided PCI strategy. A nonsignificant increase in TVF was observed in the S-iFR group.

## 1. Introduction

Intracoronary physiological assessment to guide the need for revascularization is a valuable strategy strongly recommended by the clinical guidelines [1]. Unlike fractional flow reserve (FFR), the nonhyperemic index instantaneous wave-free ratio (iFR) allows for the evaluation of the physiological contribution of each lesion or coronary segment in patients with tandem lesions or diffuse coronary disease [2]. This has allowed the development of a specific software, the Syncvision software (version 4.1.0.5, Philips Volcano, Belgium), that translates the physiological contribution of each segment visually in the form of yellow dots [3,4]. Furthermore, the software provides an estimation of the expected physiological benefit after the percutaneous coronary intervention (PCI).

However, there is limited data on the application of this software and the potential benefits of adopting this strategy in routine clinical practice to guide PCI decision-making in terms of planning and post-PCI functional evaluation [5]. It is generally assumed that a good angiographic result after PCI is equivalent to a good physiological result. However, the uncertainty surrounding this assumption has been reflected in a significant number of studies with post-PCI physiological evaluation [6,7,8,9].

We hypothesize that a routine physiological PCI guidance strategy with SyncVision/iFR (S-iFR) in patients with sequential and/or diffuse coronary artery lesions (and distal positive iFR) will avoid unnecessary stenting as compared to a standard angiography-guided PCI strategy while resulting in a good physiological result [10].

## 2. Materials and Methods

### 2.1. Trial Design

We conducted an investigator-initiated multicenter, randomized, controlled, and open-label trial to compare S-iFR-guided revascularization to angiography-guided revascularization in patients with sequential and/or diffuse significant angiography coronary lesions. The study protocol has been published previously (ClinicalTrials.gov identifier: NCT04283734) [10]. The study received the proper ethical oversight and was approved by the local Ethical Committee of Córdoba. All the patients provided written informed consent. This was an independent trial with no financial support from Philips Volcano.

### 2.2. Participants

Patients with chronic or acute coronary syndrome (nonculprit vessel) undergoing PCI at Reina University Sofía Hospital (Córdoba, Spain) and Leon University Hospital (León, Spain) February 2020 and February 2023 were eligible for enrollment if they were older than 18 years, they presented a vessel with sequential lesions separated by <10 mm from each other with a total lesion length ≥ 25 mm and a percent diameter stenosis >60% in, at least, one segment; or a coronary segment with diffuse disease higher than 30 mm and diameter stenosis >60% who presented a distal iFR ≤ 0.89. As exclusion criteria, we established the following: Acute Coronary Syndrome with nonoptimal results at the culprit vessel (final TIMI flow < TIMI III, nonreflow phenomenon during the treatment, residual coronary dissection and/or loss or compromise of a significant side branch); left ventricular ejection fraction lower than 45%; life expectancy < 12 months; coronary anatomy more suitable for surgical revascularization after Heart Team discussion; severe aortic stenosis; and contraindication for dual antiplatelet therapy during at least 12 months. There were no eligibility criteria based on the characteristics of the target vessel; the need for revascularization was based on the operator criteria.

Eligible patients were randomly assigned in a 1:1 ratio to undergo S-iFR-guided PCI or angiography-guided PCI. Randomization was performed centrally with a web-based system using four random block sizes. The allocation sequence was concealed from investigators and patients.

### 2.3. Interventions

After angiographic evaluation, vessels with long, tandem, or diffuse coronary lesions were evaluated by iFR to assess the physiological significance of the lesions. If the distal iFR was ≤0.89 and the patient accepted to participate in the study, two expert operators determined the coronary segment to treat in case the patient was allocated to angiography-guided PCI (based on angiography stenosis, lesion length, lesion characteristics, landing zone, and angiography-PCI experience). After that, the patient was randomized. The use of intracoronary imaging was permitted in both groups to optimize the angiographic result and was left at the operator’s discretion.

Patients randomized to angiography-guided PCI were treated according to previous decisions by expert operators. The treatment of additional lesions or segments was left to operator criteria if the final result was not optimal (angiography or intracoronary imaging).

In patients randomized to S-iFR, the operator performed a baseline iFR-pullback to establish the revascularization strategy. The flowchart of the technical treatment has been previously detailed [10]. In summary, if the iFR improvement was diffuse, without accumulation of dots (of <20% of the total number of dots), the vessel was not revascularized. If there was an accumulation of dots, the operator treated the minimum segment to achieve an iFR > 0.89. After that, a new iFR pullback was performed to confirm the result, optimizing the result if necessary (Supplementary Data Figure S1).

### 2.4. Endpoints

The primary endpoint was the total stent length implanted at the target vessel. Secondary endpoints included target vessel failure (TVF) at one year, definitive or probable stent thrombosis, target lesion revascularization, procedural time, contrast dose, and radiation exposure. TVF was defined according to the Academic Research Consortium-2 Consensus Document on Standardized End Point Definitions for Coronary Intervention Trials and included cardiovascular death, myocardial infarction, and target vessel revascularization) [11]. Although the initial protocol included the systematic evaluation of residual ischemia with single-photon emission computed tomography at the 6-month follow-up, the protocol was amended, and the scan was only performed if residual angina symptoms were present.

### 2.5. Follow-Up

Clinical follow-up was performed at 3, 6, and 12 months after the PCI procedure (in-person or via phone calls).

### 2.6. Statistical Analysis

For the primary endpoint, we estimated that a sample size of 50 participants in each group would provide the trial with 90% power to detect an effect size of 0.65 (15 mm difference in stent length) at a two-sided alpha level of 0.025.

Continuous variables were summarized as mean ± standard deviation or mean (P25-P75), and qualitative variables as absolute numbers and percentages. The difference in the stent length (the primary endpoint) was compared between the groups with a two-sample unpaired *t*-test. To compare continuous variables between the groups, the Student *t*-test was also used, while the chi-squared test was used to compare qualitative variables. To evaluate the risk of TVF and other clinical outcomes, time-to-event analyses were conducted using Kaplan–Meier curves and Cox proportional-hazards models. In all cases, differences were considered significant with *p* values < 0.05. R version 4.3.2 was used for the analysis.

## 3. Results

### 3.1. Baseline Characteristics

**Figure 1:**
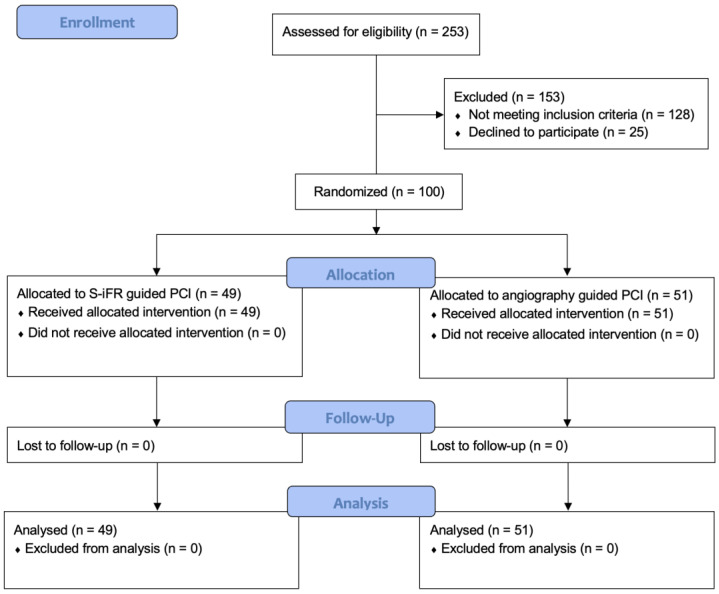
CONSORT diagram of participant flow. PCI: Percutaneous coronary intervention. S-iFR: Syncvision/iFR.

During the recruitment period (February 2020 to January 2023), 100 subjects who met the eligibility criteria were enrolled in the study. Of the 100 patients, 49 were assigned to the Syncvision-guided group and 51 to the angiography-guided group (Figure 1).

The baseline demographic and clinical characteristics of the patients were well-balanced between the groups, and are shown in Table 1. The mean age of the patients was 68.2 ± 10 years; 77% were male, and 42% presented with stable angina. The target lesions and procedural characteristics of the two trial groups are shown in Table 2.

The target vessel did not differ significantly between groups. In both cases, the most frequent vessel included was the LAD and the most frequent type of lesions were tandem lesions followed by diffuse lesions (Table 2). The baseline mean iFR was comparable between the groups (0.67 ± 0.19 vs. 0.71 ± 0.16, *p* = 0.279).

All patients completed the assigned procedure and the follow-up, and no cross-over between groups was reported.

### 3.2. Primary and Secondary Endpoints

The implanted stent length was 32.7 ± 17.2 mm in the S-iFR group and 43.1 ± 14.9 mm in the angiography group (mean difference, −10.4 mm; 95% confidence interval [CI], −16.9 to −4.0; *p* = 0.002) (Table 3). The result was consistent after excluding the two S-iFR patients who did not undergo PCI according to the protocol (Supplementary Data Table S2). Treatment strategy and lesion length were the only predictors of implanted stent length (Supplementary Data Table S1). Regarding the angiography consensus preceding randomization, patients allocated to the S-iFR strategy showed a reduction in the implanted stent length compared to the estimated stent length (−10.7 mm, 95% CI −14.5 to −6.9, *p* = 0.001). Of note, 14 patients (30%) in the S-iFR group needed additional stent implantation regarding the first software estimation.

In the S-iFR group, the final iFR was 0.91 ± 0.04. The operators could not achieve a final iFR > 0.89 in seven patients (14%), even after optimization. All these coregistries showed a diffuse increase of iFR without further potential for improvement. Despite presenting angiographically significant lesions, two patients showed an iFR pullback indicative of diffuse disease (without dots accumulation). These patients were deferred for PCI.

No differences between the S-iFR and angiography groups were found in terms of procedural time (92 (70–105) vs. 87 (70–106) min, *p* = 0.750), fluoroscopy time (20 (6–31) vs. 17 (9–25) min, *p* = 0.442), contrast dose (178 ± 56 vs. 177 ± 70 mL, *p* = 0.941), and radiation exposure (155 ± 81 vs. 141 ± 81 Gy/cm^2^, *p* = 0.440).

**Figure 2:**
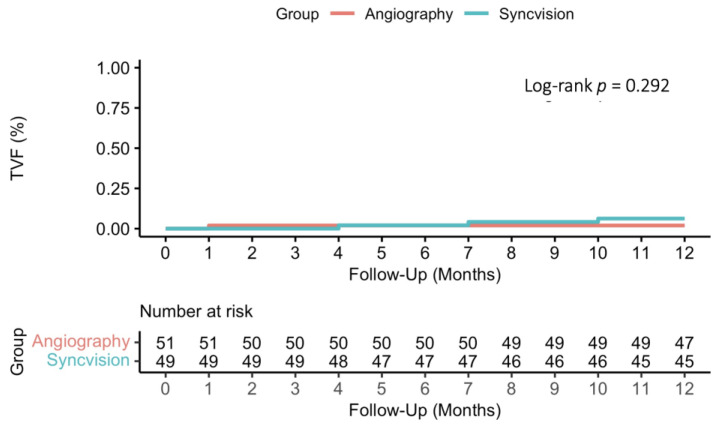
Kaplan–Meier survival analysis of one-year target vessel failure according to treatment group. TVF: target vessel failure.

At 12 months, TVF occurred in four patients: 3 (6.1%) patients in the S-iFR group, and one (1.9%) patient in the angiography group (HR 3.16, 95% CI 0.33–30.37, *p* = 0.319). This was consistent in patients with acute and chronic presentation (*p* for interaction 0.866). A Kaplan–Meier plot is shown in Figure 2, and detailed rates of the individual components of the endpoint are shown in Table 4.

**Figure 3:**
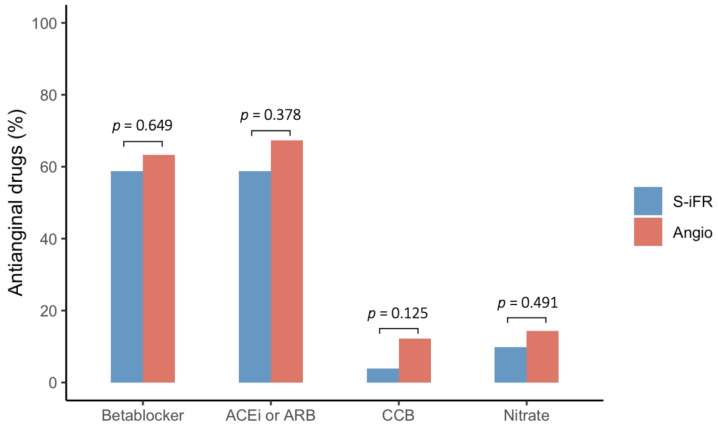
Antianginal medication. ACEi: Angiotensin-converting enzyme inhibitor, ARB: Angiotensin receptor blocker, CCB: Calcium channel blocker.

Finally, during the follow-up, only two patients, who belonged to the angiography group, presented residual angina (2 (4%) vs. 0 (0%), *p* = 0.494). There were no differences regarding antianginal medication use (Figure 3).

### 3.3. Safety

There were no intraprocedural complications in the S-iFR group and only one complication (coronary dissection) in the angiography group (0.0% vs. 2.0%, *p* = 0.999). Periprocedural myocardial infarction occurred in 2 (4.1%) and 4 (7.8%) patients in the S-iFR and angiography group, respectively (*p* = 0.457).

## 4. Discussion

The iLARDI trial showed that in patients with sequential, long, or diffuse lesions and a distal iFR ≤ 0.89, a systematic S-iFR strategy resulted in a shorter stent length implantation than an angiography-guided strategy. This strategy did not result in a difference in terms of TVF, residual angina, or the use of antianginal drugs at 12 months follow-up.

The use of intracoronary physiology to guide the need for revascularization in patients with stable angina and intermediate lesions is strongly recommended by the European Guidelines due to their clear benefit shown in multiple trials [1,12,13,14,15,16]. The two indexes with clinical evidence are the fractional flow reserve (FFR) and the instantaneous wave-free ratio (iFR) [12,13,14,15]. The first one, a hyperaemic index, was the first index that showed a correlation between the presence of ischemia by noninvasive techniques and a cut-off value of 0.80 [16]. Their utility in clinical practice was demonstrated further [13,14]. More recently, the iFR is a nonhyperaemic index that analyzes the pressure differences in a specific diastole period and resting conditions [17,18,19,20,21]. The first studies that compared both indexes established a grey zone in the range of iFR between 0.86 and 0.93 [18]. However, the main trials that confirmed the equivalence of the iFR and the FFR used a cut-off value of 0.89 that could explain the differences in the percentage of LAD revascularization between groups [15,16,22]. In our experience, the LAD has a physiological decrease in pressure. This translates into a distal iFR lower than one in arteries without epicardial lesions. Although the cut-off value is the same as other arteries, in our opinion, this physiological condition could induce an overtreatment without an improvement in the distal iFR. Fortunately, the iFR can evaluate the specific contribution of each lesion or segment [3,4]. This property is specific to this index due to the stability of the coronary flow in resting conditions. As long as the flow remains stable, the diastolic pressure changes are related to the severity of the stenosis, allowing prediction of the contribution of each lesion to the pressure change. In contrast, the FFR is unable to evaluate more than one lesion due to the coronary flow changes related to induced maximum hyperemia. In this particular condition, the coronary flow undergoes changes even with minimal stenosis. Thus, it cannot be determined whether pressure changes are attributable to flow changes or vessel stenosis, potentially leading to an underestimation of the severity of distal lesions [2].

These characteristics have permitted the creation of a specific software that has established a correlation between the angiogram and the physiology. The most important theoretical advantage of the Syncvision software is the possibility to distinguish the lesions that produce or do not produce ischemia; the differentiation between focal disease (with a clear benefit for PCI) and diffuse disease, and the prediction of the result after PCI [3,4].

To our knowledge, no randomized clinical trials have analyzed its potential benefit compared to the classical angiography-guided strategy [23,24]. Our manuscript is the first study demonstrating that a routine physiological guidance strategy during the PCI decision-making process in patients with sequential and/or diffuse coronary artery lesions (and distal positive iFR) permits treating only physiologically significant lesions, avoiding stenting of unnecessary lesions regarding a standard angiography-guided strategy. This strategy resulted in a shorter stent length implantation without significant differences in MACE, procedure parameters, or angina at 12 months follow-up.

The other randomized study analyzing the utility of an S-iFR strategy is the DEFINE GPS, which is currently in the recruitment phase.

This reduction in the stent length implantation is related to a reduction in the number of lesions treated in the S-iFR arm, confirming that the routine use of this software permits avoiding overtreatment of intermediate coronary lesions. It is known that the stent length, and probably the overtreatment, has a direct correlation with the presence of MACE at follow-up [25,26]. Until the publication of the DEFINE GPS, the main clinical implication of the iLARDI was the utility of the Syncvision-iFR software (version 4.1.0.5, Philips Volcano, Belgium) for PCI decision-making. This strategy permits the establishment of an objective approach based on iFR pullback, the treatment of only lesions with physiological impact, the confirmation of a good physiological result afterward, and the reduction of the number and length of stents implanted in patients. For this reason, the DEFINE GPS will confirm if these promising results present advantages in terms of MACE reduction at follow-up.

Recently, the 5-year follow-up of the iFR SWEDHEART and DEFINE FLAIR trials have been reported [27]. In contrast with other subanalyses, the iFR group presented a higher incidence of all-cause mortality. Authors speculate it could be associated with a higher percentage of nontreated lesions at distal left main and ostial LAD. However, there is no data on the anatomical coronary characteristics and the cause of death. For this reason, these data should be interpreted with caution.

This study has potential limitations that should be acknowledged. First, the open-label design since the operators cannot be masked to the revascularization strategy. Second, the limited sample size is powered for the primary outcome but not for clinical events. Additionally, the limited follow-up time prevents a long-term assessment of the incidence of clinical events or symptoms. Finally, when the study protocol was designed, there was no robust data on the impact of a final iFR < 0.95 after PCI, and the protocol established an objective of an iFR > 0.89.

## 5. Conclusions

Among patients with sequential or long, diffuse coronary lesions, a routine Syncvision-guided strategy resulted in a reduction of the global stent length compared to an angiography-guided strategy. However, this strategy was associated with a numerical increase in target vessel failure. Larger studies are needed to evaluate the clinical significance of these findings.

## Figures and Tables

**Table 1:** Clinical data.

	S-iFR n = 49	Angiography n = 51	*p*
Male sex	37 (75.5)	40 (78.4)	0.729
Age (years)	69.0 ± 9.5	67.5 ± 10.0	0.435
Hypertension	35 (71.4)	28 (54.9)	0.087
Diabetes mellitus	22 (44.9)	16 (31.4)	0.164
Dyslipidemia	31 (63.3)	29 (56.9)	0.514
Smoker	8 (16.3)	18 (35.3)	0.031
Prior coronary artery disease	10 (20.4)	9 (17.6)	0.725
Previous revascularization			0.999
Percutaneous	13 (92.9)	10 (100)	
Surgical	1 (7.1)	0	
Atrial fibrillation	2 (4.1)	4 (7.8)	0.678
Heart failure	7 (14.3)	4 (7.8)	0.303
Prior stroke	2 (4.1)	4 (7.8)	0.678
Peripheral atherosclerosis	5 (10.2)	3 (5.9)	0.483
Previous major bleeding	1 (2.0)	0	0.490
Hemoglobin (mg/dL)	13.7 ± 1.7	14.1 ± 1.7	0.264
Creatinine (mg/dL)	0.9 (0.8–1.1)	0.9 (0.8–1.1)	0.894
LVEF (%)	59 (50–60)	60 (49–65)	0.591
Clinical presentation			0.837
Stable angina	22 (44.9)	20 (39.2)	
Unstable angina-NSTEMI	16 (32.7)	19 (37.3)	
STEMI	11 (22.4)	12 (23.5)	

LVEF: Left ventricular ejection fraction, NSTEMI: non-ST-elevation myocardial infarction, S-iFR: Syncvision/iFR-guided percutaneous coronary intervention strategy, STEMI: ST-segment elevation myocardial infarction.

**Table 2:** Procedural data.

	S-iFR n = 49	Angiography n = 51	*p*
Radial access	49 (100)	50 (98)	0.999
Multivessel disease	27 (55)	34 (67)	0.236
Syntax score	15 (9.5–24)	15 (10–21)	0.858
Number of vessels to revascularize	1 (1–2)	2 (1–2)	0.090
Randomized vessel			0.117
LAD	34 (69)	35 (69)	
LCx	3 (6)	9 (18)	
RCA	12 (25)	7 (14)	
Other	0 (0)	0 (0)	
Type of lesion			0.491
Tandem lesion	42 (86)	46 (90)	
Long diffuse	7 (14)	5 (10)	
Vessel reference diameter	2.5 (2.5–3)	3.0 (2.5–3)	0.034
Vessel stenosis	80 (70–80)	75 (70–80)	0.399
Vessel MLD (mm)	0.6 (0.5–0.9)	0.8 (0.5–0.9)	0.055
Vessel lesion length (mm)	42.3 ± 12	39.8 ± 12	0.297
Estimated stent length (mm)	43.4 ± 13.5	41.0 ± 12.9	0.401
Stent diameter (mm)	2.5 (2.5–3.0)	2.75 (2.5–3.0)	0.029
Baseline iFR	0.71 ± 0.16	0.67 ± 0.19	0.279
Procedural time (min)	92 (70–105)	87 (70–106)	0.750
Fluoroscopy time (min)	20 (6–31)	17 (9–25)	0.442
Radiation (Gy/cm^2^)	155 ± 81	141 ± 81	0.440
Contrast (mL)	178 ± 56	177 ± 70	0.941
Intraprocedural complications	0 (0)	1 (2)	0.990
Periprocedural myocardial infarction	2 (4.2)	4 (7.8)	0.68
Peak of troponin (ng/mL)	0.9 (0.1–16.3)	3.1 (0.3–22.6)	0.177
In-hospital complications			N/A
Bleeding	0 (0)	0 (0)	
Death	0 (0)	0 (0)	
Stroke	0 (0)	0 (0)	
Stent thrombosis	0 (0)	0 (0)	
Intracoronary imaging	1 (2.0)	5 (9.8)	0.205
Rotablation	0 (0)	0 (0)	N/A
Angiographic success	49 (100)	51 (100)	N/A

LAD: Left anterior descending coronary artery, LCx: left circumflex coronary artery, S-iFR: Syncvision/iFR-guided percutaneous coronary intervention strategy. N/A: not applicable.

**Table 3:** Procedural endpoints.

	S-iFR n = 49	Angiography n = 51	Difference (CI 95%)	*p*
Primary endpoint				
Implanted stent length (mm)	32.7 ± 17.2	43.1 ± 14.9	−10.4 (−16.9 to −4.0)	0.002
Other endpoints				
Difference implanted—estimated (mm)	−10.7 ± 13.2	2.1 ± 7.7	−12.8 (−17.2 to −8.5)	0.001
Final iFR	0.91 ± 0.03	-	-	-
Estimated stent length by Syncvision to achieve an iFR > 0.89 (mm)	29.2 ± 14.0	-	-	-
Dots accumulation to predict an iFR improvement	47 (94)	-	-	-

S-iFR: Syncvision/iFR guided percutaneous coronary intervention strategy.

**Table 4:** Clinical endpoints.

	S-iFR n = 49	Angiography n = 51	Hazard Ratio or Risk Difference (CI 95%)	***p***
Clinical secondary endpoints				
Target vessel failure	3 (6.1) *	1 (1.9)	3.16 (0.33 to 30.37)	0.319
Cardiovascular death	0 (0)	1 (1.9)	−1.9 (−5.9 to 1.9)	0.322
Myocardial infarction	2 (4.1)	0 (0)	4.1 (−1.7 to 9.8)	0.159
Stent thrombosis (definite or probable)	0 (0)	0 (0)	-	-
Target vessel revascularization	2 (4.1)	0 (0)	4.1 (−1.7 to 9.8)	0.159
Target lesion revascularization	2 (4.1)	0 (0)	4.1 (−1.7 to 9.8)	0.159
Stroke	1 (2.0)	1 (1.9)	0.0 (−5.5 to 5.7)	0.977

S-iFR: Syncvision/iFR-guided percutaneous coronary intervention strategy. The hazard ratio is shown for target vessel failure and risk difference for the other endpoints. * None of the events occurred in the two patients with untreated diffuse disease.

## Data Availability

The data collected for this trial can be made available to others upon reasonable request to the corresponding author.

## Supplementary Materials

The following supporting information can be downloaded at: https://www.mdpi.com/article/10.3390/jcm13154342/s1, Figure S1: Flowchart of technical treatment details of patients randomized to the S-iFR group; Table S1: Predictors of implanted stent length. Linear regression models.; Table S2: Primary endpoint analysis excluding two S-iFR patients with diffuse disease (no stent implantation).
