# Supplementary material for: The Usefulness of Coregistration with iFR in Tandem or Long Diffuse Coronary Lesions: The iLARDI Randomized Clinical Trial
Source: J Clin Med. 2024 Jul 25;13(15):4342. doi: 10.3390/jcm13154342 (PMC11313554; doi:10.3390/jcm13154342)
Supplement: Supplementary file 1 [file jcm-13-04342-s001.zip › jcm-3093638-supplementary.pdf]

Table S1. Predictors of implanted stent length. Linear regression models.

	Univariable			Multivariable		
	Beta	95% CI	P-value	Beta	95% CI	P-value
Number of vessels treated	-0.39	-5.54, 4.75	0.878			
Vessel reference diameter	-2.87	-11.51, 5.75	0.510			
Vessel stenosis (percentage)	-0.15	-0.50, 0.18	0.368			
Vessel lesion length	0.97	0.77, 1.17	<0.001	1.039	0.87, 1.20	<0.001
LAD	-0.50	-7.78, 6.76	0.890			
Tandem lesion	-3.33	-13.65, 6.98	0.523			
S-iFR	-10.45	-16.86, -4.03	0.002	-13.38	-17.44, -9.31	<0.001

CI: confidence interval, LAD: Left anterior descending coronary artery, S-iFR: Syncvision/iFR guided percutaneous coronary intervention strategy

Table S2. Primary endpoint analysis excluding two S-iFR patients with diffuse disease (no stent implantation)

	S-iFR<br>n=47	Angio<br>n=51	Difference (CI 95%)	p
Primary endpoint<br>Implanted stent length (mm)	34.1 ± 16.1	43.1 ± 14.9	-9.03 (-15.31 to -2.75)	0.005

S-iFR: Syncvision/iFR guided percutaneous coronary intervention strategy; CI: confidence interval

Figure S1. Flowchart of technical treatment details of patients randomized to the S-iFR group

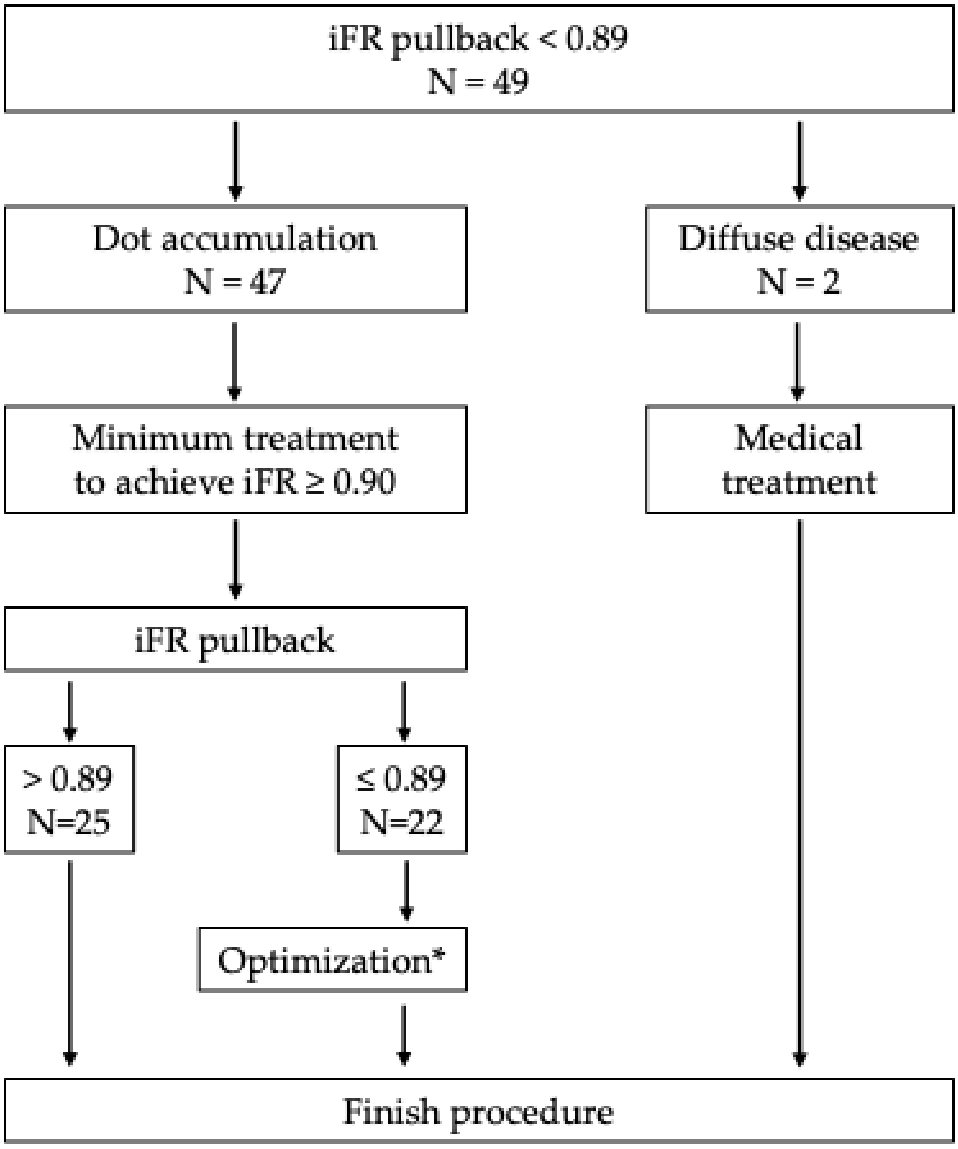

iFR > 0.89 not achieved in 7 patients after optimization (no further dot accumulation)
